# Autologous natural killer cells as a promising immunotherapy for locally advanced colon adenocarcinoma: Three years follow‐up of resectable case

**DOI:** 10.1002/cnr2.1866

**Published:** 2023-07-13

**Authors:** Budhi Ida Bagus

**Affiliations:** ^1^ Department of Surgery Sebelas Maret University Surakarta Indonesia

**Keywords:** autologous natural killer cells, colon adenocarcinoma, locally advanced

## Abstract

**Background:**

Over the last decade, a new modality of immunotherapy has been announced, with the expectation of better long‐term clinical outcomes and disease‐free survival after the definitive surgical treatment of colon cancer. Natural killer (NK) cells as part of cellular therapy in immunotherapy have the potential effect as an adjuvant therapy for locally advanced and metastasized colorectal adenocarcinoma. We would evaluate the clinical outcome of autologous NK cell therapy for resectable colon cancer.

**Case:**

A 64‐year‐old woman presented with a transverse colon tumor‐related partial intestinal obstruction and a history of bloody diarrhea. A transverse colectomy has been done, and the pathology report reported adenocarcinoma of the transverse colon and positive lymph node involvement (TNM stage III). The patient had R0 resection status. A PET scan was done 6 months later, with positive lymph node glucose uptake at mesocolic. NK cell therapy was administered for 2 cycles with a 3‐month interval, and PET scan follow‐up was done 3 years after resection; no more glucose uptake was found, and the patients tolerated the therapy well with no immune‐related adverse effects reported.

**Conclusion:**

As a new modality in immunotherapy strategies for locally advanced colon adenocarcinoma, particularly in cases unsuitable for standard chemotherapeutic treatment, autologous NK cells have a promising effect and are feasible and well tolerated in our clinical practice.

## 1 INTRODUCTION

The potential of immunotherapies based on natural killer (NK) cell involvement was first discovered in the hematological setting. However, this discovery has spurred the construction of many immunological responses that can also be used against solid tumors, such as colorectal cancer (CRC). In point of fact, despite efforts toward cancer prevention in the form of screening programs, surgical procedures, and chemotherapy, CRC remains one of the malignancies with the highest mortality rate.

In recent years, there has been an increase in interest in using the NK anti‐tumor capacity as a novel immunotherapeutic strategy, and various clinical trials based on NK cell infusion have been designed or are being planned.^1^ Some of these studies are currently underway, such as a phase I study in which the effectiveness of allogeneic, in vivo activated NK cells is assessed in several advanced solid tumors, including CRC, either alone or in combination with monoclonal antibodies (trastuzumab or cetuximab).^1^, ^2^

Drug development has exploded in response to immunotherapy's potential to induce long‐lasting remissions in advanced diseases, with a particular focus on creative combinatorial immunotherapeutic approaches. NK cells as part of cellular therapy in immunotherapy have the potential as an adjuvant therapy for locally progressed and metastatic colorectal adenocarcinoma.^3^

With the creation of numerous immune cells that can be used as therapeutic agents, cancer immunotherapy has firmly established itself as a new benchmark in cancer treatment. NK cells are innate immune cells with strong cytolytic activity against tumors that also serve as immune system regulators. Immune stimulants including cytokines and antibodies, as well as adoptive transfer of activated NK cells generated ex vivo, can improve the effectiveness of NK cell‐mediated immunotherapy.^2^, ^3^

Particularly in our developing country with many limitations on advanced cancer treatment support from the government health insurance and private insurance, the routine guideline that we use is adjuvant chemotherapy for locally advanced CRC. As a brand‐new cellular therapy, usually we use these as additional treatments after standard chemotherapy or a combination of them. We lack supporting data in our local cases, which can be used as an option for locally advanced CRC. Although many prospective cohort studies reported the effectiveness of this cellular therapy on reduced the cancer progression and having good clinical outcomes (disease‐free survival [DFS]).^4^

Natural killer cells are supportive therapies in cancer therapy and other immune disorders that make use of the body's immune cells, namely T cells, NK cells, and other cells. These cells naturally exist in our body and are useful to attack cancer cells either directly or indirectly.^5^

## 2 CASE REPORT

We would evaluate the clinical outcome of autologous NK cell therapy on resectable colon cancer; this case was presented from the Surgery Department of Moewardi General Hospital, Indonesia on December 11, 2018.

A 64‐year‐old woman presented with a transverse colon tumor‐related partial intestinal obstruction and a history of bloody diarrhea. From the family history, the patient has no history of related cancer in her first or second‐degree relatives. No history of tumors in other organs related to her family. Preoperative carcino embryonic antigen (CEA) level was 1.5 ng/mL (<3.0 ng/mL), and the patient has no cardiopulmonary comorbidity. The pre‐operative laboratory examination was within the normal limit. The hemoglobin level was 11.7 g/dL (12.0–15.6 g/dL), the white blood count was 10 300/uL (4500–110 000/uL), the platelet count was 146 000 /uL (150 000–450 000 /uL), the urea level was 45 mg/dL (<50 mg/dL), the creatinine level was 0.8 mg/dL (0.6–1.1 mg/dL), and the aspartate aminotransferase was 18 IU/L (4–40 IU/L), alkaline phosphatase was 57 IU/L (20–140 IU/L).

On December 2018, Laparotomy‐transverse colectomy has been done, and the pathology result reported adenocarcinoma of the transverse colon and positive lymph node involvement (stage III). The patient had R0 resection status. Two months after surgery, the follow‐up CEA level was 1.3 ng/mL.

In this case study, we did not manage the adjuvant chemotherapy because of the history of multiple allergenic cases that led to Steven Johnson syndrome a decade before and because the patient refused to take these adjuvant chemotherapy treatments. A PET scan was done 6 months later, showing positive lymph node glucose uptake in the mesocolic and peritoneal regions, signed by the arrow marks.

(Figure 1).

**FIGURE 1:**
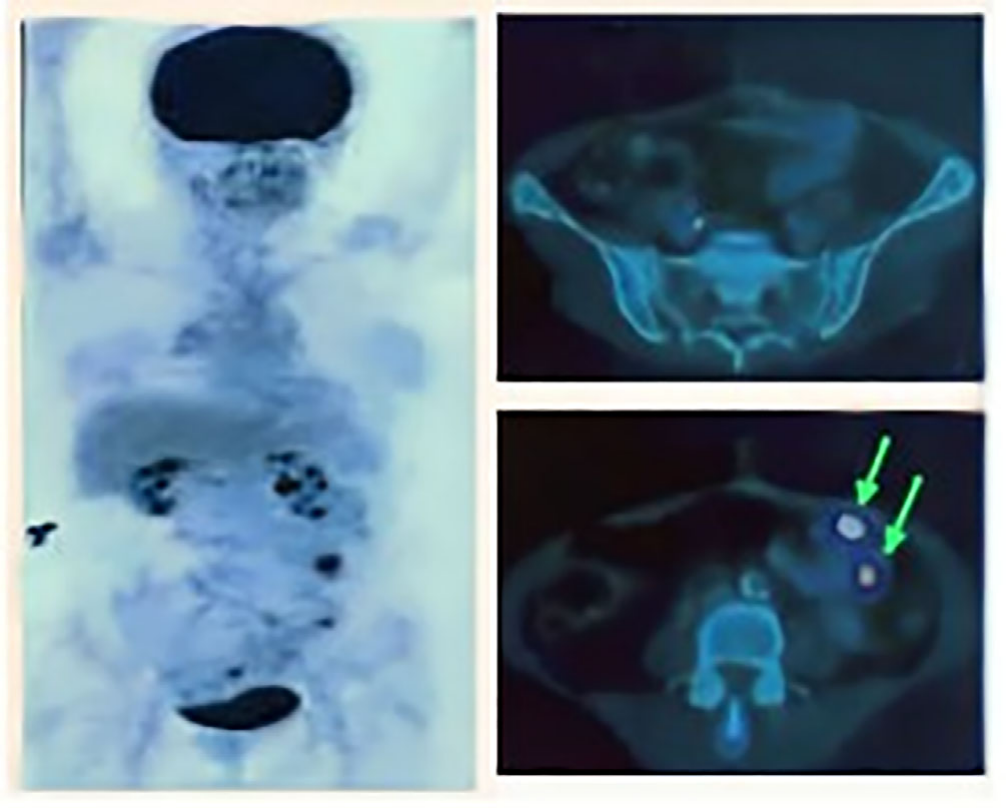
Axial view of abdominal positron emission tomography scanning showing the fluoro‐deoxy‐glucose uptake in the peritoneum and mesocolica region outside the intraluminal of the colon indicated by a light arrow marks.

Natural killer cells therapy preparation, increasing the immune system of the patient by multiplying the number of immune cells from the patient's body (autologus), then activating, and infusing them back into the patient's body. This cellular therapy begins by taking approximately 60 cc of patient peripheral blood, followed by a process of reproduction and activation for 2 weeks, and then infused back to the patient for about one and a half hour.

**FIGURE 2:**
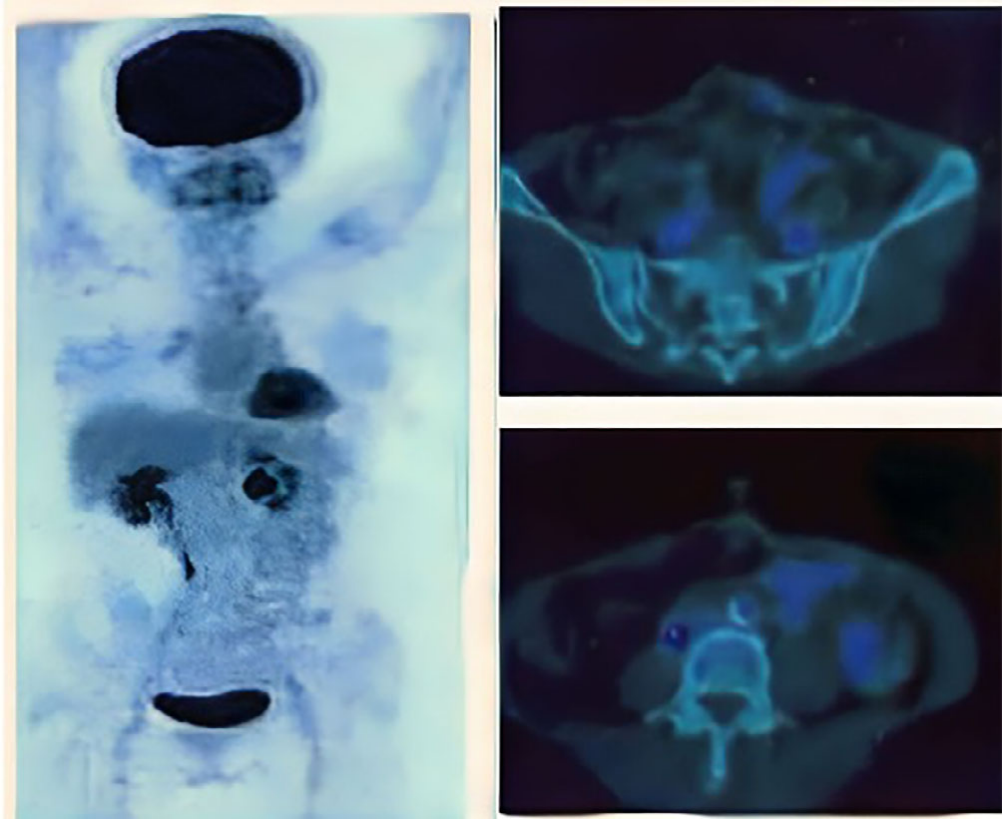
Positron emission tomography‐scan evaluation after natural killer cells treatment, on the axial view we found no more fluoro‐deoxy‐glucose uptake in the locoregional metastasis and no additional uptake in the intra‐abdominal cavity.

Natural killer cells therapy has administered for 2 cycles, with an interval of 3 months for the second treatment and PET scan follow‐up was done 3 years after resection; no more glucose uptake was found, and the patients tolerated the therapy well with no immune‐related adverse effects reported (Figure 2). During clinical follow‐up 3 years after the definitive surgery for the colon cancer, the patients could tolerate their daily activities well; there were no signs and symptoms of locoregional recurrence and no long‐term cardio‐pulmonary morbidity until late December 2022.

## 3 DISCUSSION

The CEA level was 1.5 ng/mL (the normal level is 3.0 ng/mL). This biomarker examination was our routine pre‐operative evaluation for CRC. Although it does not play a role in the progression of the cancer, we still use this marker to follow‐up on whether there are any possibilities of local or distant metastasis if we find an increasing of the CEA level on the post‐operative period.

According to the TNM stage, this case was a transverse colon adenocarcinoma stage III. Our national CRC guidelines recommend administering the adjuvant FOLFOX regimen (5‐fluorouracyl, leucovorin, and oxaliplatin) chemotherapy infusion at a 2‐weekly interval for 6 months, as suggested by the latest NCCN guidelines for colon and rectal cancer. The other treatment option was immunotherapy as a single adjuvant or in combination with routine chemotherapy. In this case study, the patient had a previous history of multiple allergic reactions and Stevens‐Johnson syndrome, so we decided to administer these cellular therapies as a single therapy.

As supportive therapies in cancer therapy, particularly in CRC, NK cells and other immune cells, namely T cells, both have an important role in reducing tumor progression and invasion. Peritoneal and paracolica tumor infiltration, which was found on the PET scan, indicated that our routine post‐operative evaluation by using an abdominal CT scan still has limitations, even in a stable clinical condition of the patient, although current guidelines do not recommend the PET scan as a routine modality for evaluating the local or distant metastasis of these cancers.^4^

Natural killer cells are cells derived from lymphocytes that play an important role in the immune system to fight cancer and viruses. These cells are also called NK cells because they can act immediately without the need for activation. Target cells (cancer or virus‐infected cells) will suffer death. The number of NK cells in the body is only about 10%–15% of all lymphocytes in the blood. And this number will be reduced in patients with immune deficiency diseases such as cancer, viral infections.^3^, ^5^ The basic principle of cellular therapy particularly in CRC patients is by increasing the NK cell level in the body of the patients and the further process is the increasing cytotoxic effect from this therapy.

Natural killer cells have the potential to significantly reduce tumor progression and recruit additional immune subpopulations without the need for prior antigen presentation, unlike T or B cells, which may require removal of endogenous antigen specificity mediated via the T cell receptor (TCR) and/or the B cell receptor (BCR). In the fight against cancer, NK cells have emerged as a key component of the immune response in recent years.

Early‐stage CRC has good long‐term survival; definitive surgery could control the tumor and locoregional metastasis, which has 90% of 5‐year survival. On the contrary, for advanced stages of CRC, 5‐year survival decreased by only 13% with additional routine adjuvant treatment such as radiotherapy or chemotherapy. In our case study, although the patient received no adjuvant treatment as a routine protocol, the first 3 years of follow‐up represented a favorable clinical outcome, with no sign or symptom of an immune‐related adverse effect reported during this 3‐year period.

By using cellular therapy as a monotherapy or in combination with our standard chemotherapy regimens, the short and long‐term clinical outcomes would be better, with better control of DFS after definitive surgery. Fionda et al. reported a review study that suggested the increasing level of NK cells in the patients correlated well with the better survival and clinical outcome of CRC patients. In combination with other cytotoxic lymphocytes, the presence of both CD8 T cells and NK cells is a relevant prognostic factor in CRC and is associated with better overall survival (OS).^5^ During 3 years of evaluation and follow‐up, we can evaluate the good clinical resolution of locoregional recurrence in this case in the peritoneal and mesocolica region after NK cells monotherapy treatment, although PET scans are not routinely used as a radiologic evaluation. By using this PET scan, we can detect these locoregional recurrences in the R0 resection case, which could not be easily detected on routine abdominal CT scan imaging.

In this preliminary study, we reported the effectiveness of NK cells as monotherapy (immunotherapy) for this single case of locally advanced colon cancer. As an option for our routine use of chemotherapy agents, or in combination with those agents as adjuvant treatment, the clinical efficacy of the NK cell therapy could be tolerated well. Although this kind of therapy is not routinely used in our clinical practice (especially in our developing country), according to the promising effect, the NK cell therapy would be our choice in the future. For the next step, the immune‐related adverse effect should be evaluated over a long period of time.

Immunotherapy for solid cancers, including CRC, could be administered as monotherapy or in combination with conventional chemotherapeutic agents (5‐fluorouracil, leucovorin, oxaliplatin, irrinotecan, and such targeted therapies). Many studies have reported that the combination therapy of chemo and immunotherapy gives a better clinical outcome, DFS, and OS.^6^ In this case study, the patient received only cellular therapy because she was unsuitable for adjuvant chemotherapy.

Immune‐related adverse effects during immunotherapy administration are one of many issues that should be discussed; it would be too early to state that NK cell therapy has no immune‐related adverse effects according to this report. We should evaluate the OS, including the quality of life, of the patients receiving this immunotherapy.^7^

The landscape of oncological treatment has undergone a paradigm shift thanks to cancer immunotherapy. It has become obvious that NK cells provide various advantages that can be utilized for immunotherapy, with potentially fewer side effects, in contrast to most research, which concentrated on saving T‐cells from depletion in an effort to unleash tumor‐specific immune responses.^3^, ^6^ NK cell immunotherapy as a stand‐alone therapy or in conjunction with chemotherapy would be advantageous options in this situation because chemotherapy causes resistance in many types of cancer, including CRC.^8^ This favorable response of NK cell therapy on resectable cancer has been evaluated in a case with R0 resection status and a tolerable adverse effect. We should take into consideration evaluating the other cases with R1 or R2 resection types to see if they would have the same response as this ideal R0 status.

The immune system's innate defensive mechanisms depend heavily on NK cells to eliminate various aberrant or stressed cells. NK cells are distinct from other lymphocytes in that their ability to recognize antigens is not determined by antigen specificity but rather by the integration of signals from both activating and inhibitory receptors, which are drawn to putative target cells by ligands.^9^ Without pre‐sensitization, NK cells can destroy a range of aberrant or stressed cells, and they can even target and kill cancer stem cells or stem‐like NK cells are critical components of the innate immune responses that malignancies elicit. The combination of activating and inhibiting signals is necessary for NK cells to perform their effector role.^10^ On the contrary, this study has its limitations; we have a lack of supporting data and information about the role of this cellular therapy in the unresectable cases, and whether those cases could be managed with NK cell therapy as a palliative treatment is still debatable because there are no guidelines that could support it. Although a review reported by Fionda et al suggested the presence of NK cells and other cytotoxic lymphocyte like CD8^+^ T cells could control CRC metastasis and progression.^6^

The other consideration against the role of NK cells as an immunotherapy for solid cancers, such as CRC, is the possibility of overcoming resistance to this cellular therapy.^11^ Long‐term follow‐up and further study should be done to support the wide recommendation of cellular therapy, particularly for CRC. As a clinician, what we should do is conduct closed follow‐up and clinical evaluation during this immunotherapy treatment.

Because it is known that the immune system has a role in the development and spread of CRC, certain cell subsets, such as T cells, NK cells, and natural killer T (NKT) cells, are relevant study targets for immunotherapy and clinical biomarker research. The function of systemic immune profiles in the development of tumors has been a mystery up to this point. Krijgsman et al reported that the peripheral circulating level of the NK cell is associated with these kinds of progression and clinical outcomes.^12^ Actually, that was our limitation, we hope that in the future, our center can perform this advanced examination not only evaluating the clinical response after cellular therapy administration.

The other issue to be discussed about this cellular therapy is we do not know yet how many times this therapy could be given to the patient, particularly when we found unresectable cases and already have no response for adjuvant chemotherapy before. In the future, we should evaluate the effectiveness of this cellular therapy in these selected cases, in our opinion, when there is a supportive facility and no comorbidity presence, the cellular therapy should have a recognition in our clinical judgment when the other therapy modalities did not give favorable response.

## 4 CONCLUSION

Autologous NK cells could have a promising effect, be feasible, and well tolerated as one of the multimodal treatment options in our clinical practice as a new modality in immunotherapy strategies for locally advanced colon adenocarcinoma, particularly in cases unsuitable for standard chemotherapeutic treatment.

## AUTHOR CONTRIBUTIONS

Budhi Ida Bagus has contributed on conception, design, interpretation of data and responsible for the content of this manuscript.

## CONFLICT OF INTEREST STATEMENT

The authors have stated explicitly that there are no conflicts of interest in connection with this article.

## ETHICS STATEMENT

This case study has already been approved by Health Research Ethic Committee of Moewardi General Hospital, Surakarta, Indonesia. Ethical clearance number: 127/III/HREC/2021.

## INFORMED CONSENT

The patient has already been informed about the purpose of this case study and get patient permission on using of the clinical material such as clinical finding during operation, radiological exam for the publication. The patient already signed the written informed consent for the usage of the information and clinical images for publication.

## Data Availability

Data availability statement is not applicable.
